# A translational model to determine rodent’s age from human foetal age

**DOI:** 10.1038/s41598-017-17571-z

**Published:** 2017-12-08

**Authors:** Yoshiyuki Ohmura, Yasuo Kuniyoshi

**Affiliations:** Department of Mechano-Informatics, Graduate School of Information Science and Technology, The University of Tokyo 7-3-1, Hongo, Bunkyo-ku, Tokyo Japan

## Abstract

To understand the prenatal origin of developmental and psychiatric disorders, studies in laboratory animals are imperative. However, the developmental pace differs between humans and animals; hence, corresponding human ages must be estimated to infer the most vulnerable developmental timings in humans. Because rats and mice are extensively used as models in developmental research, a correspondence between human foetal ages and rodents’ ages must be precisely determined; thus, developing a translational model is of utmost importance. Optimizing a translational model involves classifying the brain regions according to developmental paces, but previous studies have conducted this classification arbitrarily. Here we used a clustering method and showed that the brain regions can be classified into two groups. To quantify the developmental pace, we gathered data for a range of development events in humans and rodents and created a linear mixed model that translates human developmental timings into the corresponding rat timings. We conducted an automatic classification of brain regions using an EM algorithm and obtained a model to translate human foetal age to rat age. Our model could predict rat developmental timings within 2.5 days of root mean squared error. This result provides useful information for designing animal studies and clinical tests.

## Introduction

Human development is affected by several environmental factors, such as stress, nutrition and sensory inputs^1–3^. To understand the prenatal origin of human developmental disorders, studies in laboratory animals are imperative. However, the developmental pace differs between humans and other animals^4–7^; hence, corresponding human ages must be estimated to infer the most vulnerable developmental timings in humans. Because several developmental events have sensitive periods^8–10^, the precise timings are necessary to design clinical tests.

Developmental stages differ regionally between the central nervous system and body parts, as well as according to the developmental origin^5–7^. For example, in several species, animals are born before the eyelids open^4^, whereas human infants can open their eyes before birth^11^. Thus, the sequential order of development is different among mammal species, indicating that translation between animal age and human age must be calculated in relation to different body parts. Because the time-keeping mechanisms underlying the development of different body parts remain unknown, the body parts have to be classified by systematically clustering the empirical data. However, previous studies have used not only brain regions but also functional categories for classification, such as the limbic system, which included broad brain regions^5,6^.

In the present study, we classified whole brain regions into multiple groups according to their developmental pace. We surveyed the published literature on developmental events in the prenatal human and prenatal and postnatal rodent brains and made an exhaustive list of comparative developmental events. Because rodents are extensively used in developmental research, their developmental information is extensive and the translation between rodents and humans is valuable. We created a linear mixed model^12^ and selected the most plausible model using an extension of Akaike’s information criterion^13^ (EIC)^14^.

## Results

### Collection of developmental timing

We collected data on 94 developmental events with comparable timing in humans and rodents from 153 published studies (Table 1, Table S1 in Supplementary Information). The publication list was described in Table S1. Several developmental events were excluded because the cellular type and/or developmental origin, as determined by chemical cues, were not available. When discrepancies were found in published rat data, we searched for the latest experimental results in mice and surveyed the mechanism and order of the developmental events. We then selected the most consistent results according to the temporal sequence.

**Table 1:** Comparison of developmental events in humans and rats.

Developmental events	Brain region	2-class	4-class
1 First oligodendrocyte lineage in spinal cord (ventral)	Spinal cord	A2	A4
2 Gliogenetic stage in the ventral spinal cord			
3 Motor neurons expressing Er81			
4 Parvalbumin-positive fibers reach the ventral horn of the cervical segment			
5 Olig2- and Pax7-expressing cells derived from dorsal spinal cord			
6 Myelination in the cervical spinal cord			
7 Innervation of hindlimb muscle			
8 Elimination of polyneuronal innervation of hindlimb muscle			
9 PGP9.5 fibers penetrate the epidermis	DRG	B2	D4
10 Presumptive low-threshold mechanoreceptor afferent penetrates the spinal gray matter			
11 Calcitonin gene-related peptide (CGRP)-immunoreactivity in the DRG			
12 CGRP-positive fibers penetrate the epidermis			
13 Substance P-positive fibers in the taste buds			
14 CGRP-positive fibers innervate the heart			
15 CGRP-positive fibers prominent in the substantia gelatinosa			
16 Tyrosine hydroxylase (TH)-positive fibers penetrate the cortical plate	Medulla/pons	A2	B4
17 The first efferent synapse forms below the inner hair cells			
18 Axo-somatic synapses between the medial efferent and outer hair cells			
19 5-HT-positive fibers innervate the spinal gray matter			
20 First 5-HT-positive cells			
21 First appearance of noradrenergic cells			
22 Diffuse staining of Sonic hedgehog (Shh) in the inner region of the cerebellum	Cerebellum	B2	C4
23 The first IP3R1-positive cells in the Purkinje cell layer			
24 Synapse formation between climbing fibers and Purkinje cells			
25 First PV-positive Purkinje cells			
26 Shh-reactive cells disappear in the external granule layer			
27 Young climbing phase in lateral hemisphere of cerebellum			
28 TH-positive cells in the midbrain	Midbrain	A2	A4
29 Brn3a-positive cells in the ventral mesencephalon			
30 Catecholamine fibers innervate the habenula region			
31 GAP-43 expression declines in the superior colliculus			
32 TH-positive cells in the zona incerta (A13)	Thalamus	A2	A4
33 Calbindin-positive cells and processes in the anteroventral thalamus			
34 PV-positive cells in the reticular thalamus			
35 GABAergic interneurons in dorsal lateral geniculate nucleus (the dLGN)			
36 Dendrodendritic contact in dLGN			
37 Thyrotropin-releasing hormone (TRH)-positive cells in the hypothalamus	Hypothalamus	B2	D4
38 Neurophysin-positive cells in the paraventricular hypothalamus			
39 Calbindin-positive cells first appear in the lateral hypothalamus			
40 Somatostatin-positive neurons first appear in the hypothalamus			
41 Corticotropin-releasing hormone (CRH)-positive cells first appear in the hypothalamus			
42 Melatonin binding site in the suprachiasmatic nuclei			
43 Neurophysin-positive cells in the suprachiasmatic nuclei			
44 Neuropeptide-Y staining in the arcuate nucleus			
45 Calbindin-positive mammillothalamic tract fibers penetrate the ventral anterior thalamus			
46 Galamin-positive cells in the mammillary nucleus			
47 Arginine vasopressin (AVP)- staining in the suprachiasmatic nuclei			
48 Isl1-ir in the lateral ganglionic eminence	Subcortex	B2	C4
49 First acetylcholinesterase(AChE)-reactive neurons in the basal forebrain			
50 External Capsule AChE reactive			
51 AChE-positive fibers penetrate the stratum oriens in the hippocampus			
52 Myelination begin in the caudate-putamen			
53 The secondary dentate matrix forms in the hippocampus	Allocortex	B2	D4
54 Tbr2-positive Cajal-Retzius cells first appear in the hippocampus			
55 The primary germinal matrix of the dentate gyrus disappears			
56 Calbindin-positive multipolar neurons in the claustrum/amygdala			
57 Calbindin immunoreactivity in the str.lucidum along the whole CA3 region except CA3c			
58 Calbindin immunoreactivity in the str.lucidum along the whole CA3 region including the CA3c			
59 Anterior commissure fibers cross the midline			
60 Glomeruli formation in the olfactory bulb			
61 First Reelin-positive cells in the marginal zone	Isocortex	B2	C4
62 Calretinin-positive pioneer cells in the marginal zone			
63 First GABAergic neurons in the lateral cortical wall			
64 DARPP32-positive cells detected in the pallium, but not in the striatum			
65 Cortical plate formation			
66 Callosal fibers cross the midline			
67 ER81 or *Er81*-positive layer V band			
68 Npn1-positive cingulate pioneer axons			
69 Ontogeny of KCC2-positive neurons in the cortical plate			
70 Excitatory GABAergic response in cortical layer I			
71 Reelin-positive cells below the cortical surface with ascending fibers			
72 Corticospinal neurons innervate cervical spinal motor neurons			
73 Mediodorsal thalamus fibers form two intense bands in cortical layer VI			
74 Switch from bursting to acuity in the light response			
75 Radial glial processes disappear in the cerebral cortex			
76 Nrl or *Nrl* expression in the retina	Retina	A2	B4
77 Synaptophysin in the inner plexiform layer			
78 Rod opsin expression in the retina			
79 Synaptophysin in the outer plexiform layer			
80 Airways are covered with smooth muscle and enveloped by nerve trunks	Other 1	A2	
81 Open tunnel of Corti	Other 2	A2	
82 Eyes opening	Other 3	A2	
83 Gonadotropin-releasing hormone-positive cells first detected in the vomeronasal organ	Vomeronasal organ	B2	
84 Myelination begins in the optic nerve at chiasm	Optic nerve	B2	
85 Ossification of maxilla	Skeleton	A2	
86 Ossification of nasal			
87 Ossification of supraoccipital			
88 Merkel cells in the skin	Other 4	A2	
89 Nerve fibers penetrate the tongue epithelium	Other 5	A2	
90 Neuropeptide Y-positive fibers innervate the heart	Other 6	A2	
91 Onset of hair follicle bulge	Other 7	B2	
92 Onset of arrector pili muscles	Other 8	B2	
93 Eyelash growth	Other 9	B2	
94 Birth date	Other 10		

In the first column, the line in each row describes a developmental event. In the second column, the line in each row describes the related brain region. In the third and fourth columns, the line in each row describes the clustering result of two-group linear model and four-group linear model, respectively.

In contrast to a previous study^6^, six developmental events (16, 21, 52, 65, 81 and 82) overlapped. The number of developmental events identified in our human experimental dataset was larger than that in the translating time project^6^ (94 and 75 including 20 postnatal events, respectively). The number of developmental events without human data in our study was smaller than in theirs (0 and 196, respectively). Methodological differences may account for a small amount of overlap. We mainly used the onset times of chemical markers in the prenatal human brain to identify cell types. In contrast, the previous study analysed developmental changes detected by classical histological techniques in prenatal and postnatal human^6^. The strength of our dataset was the fact that developmental events without human data were not included.

Our collection of human developmental events included one *in vivo* electrophysiological analysis of a premature human infant (event 74) and one *in vitro* slice experiment (event 70). However, almost all developmental events were based on anatomical data. The developmental events were subdivided into the following: four ‘retina’, four ‘midbrain’, five ‘thalamus’, five ‘subcortex’, six ‘medulla/pons’, six ‘cerebellum’, seven ‘dorsal root/trigeminal ganglion (DRG)’, eight ‘spinal cord’, eight ‘allocortex’, eleven ‘hypothalamus’, fifteen ‘isocortex’ and thirteen others (see Methods, Table 1 and Table S1 in Supplementary Information).

### Estimation of the linear mixed model

First, we decided not to use birth-related developmental events (47 and 94) for analysis because we could not rule out the possibility that these events were outliers. A translational model of the hypothalamus using event 47 is described in Supplementary Information (Figure S1).

We estimated the linear mixed model with a categorical value [i.e. group of brain regions], using the EM algorithm^15^ to maximize the log-likelihood. We used events 1 to 79 to classify brain regions. We increased the predefined group number until EIC became the minimum. However, when the group number was greater than five, the EM algorithm did not converge because standard deviation could not be calculated due to lack of samples in the smallest group. As a result, the optimized group number was four. EIC of one-group, two-group, three-group and four-group models were 428.4, 369.0, 361.9 and 360.3, respectively. The estimation error of the translation of human foetal age to rat age was 2.4 days of root mean squared error.

**Figure 1:**
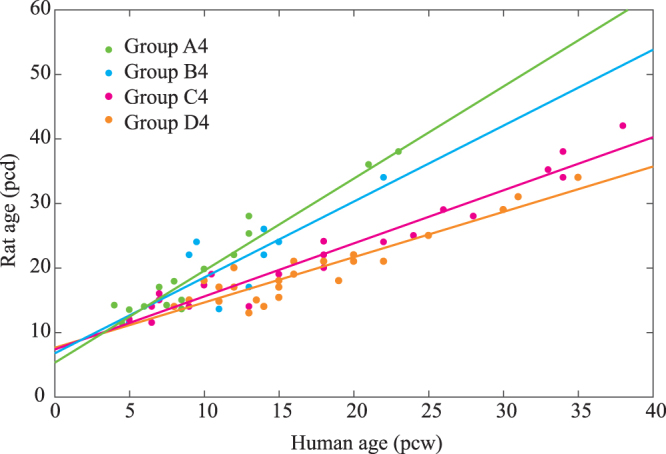
Optimized linear mixed model. Our analysis revealed that brain regions could be classified into four groups by comparing the time of development between humans and rats. The filled circles represent developmental timing. The lines represent the optimized regression line. Green, cyan, magenta and orange represent clusters of the spinal cord, medulla, cerebral cortex and hypothalamus, respectively.

The automatically determined classification of brain regions revealed that developmental events in the spinal cord, midbrain and thalamus belong to the same group. The second group consisted of the medulla/pons and retina. The third group consisted of the subcortex, isocortex and cerebellum. The fourth group consisted of developmental events in the DRG, allocortex and hypothalamus. These results are summarized in Table 1 and Fig. 1.

### Comparison of the developmental pace

**Figure 2:**
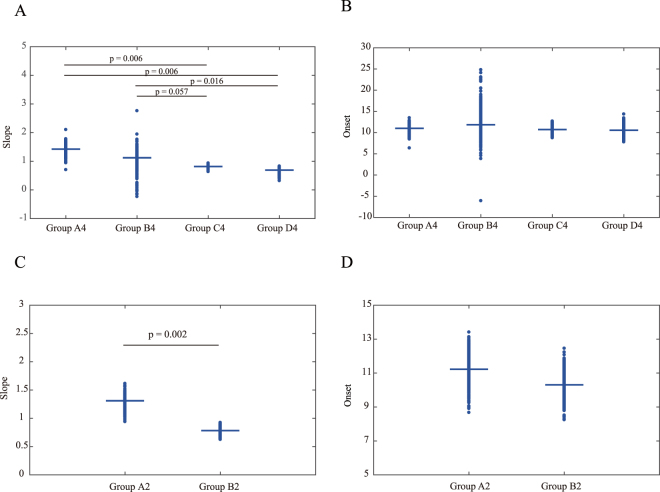
Group differences in the regression slope and developmental onset. (**A**,**C**) The developmental paces were significantly different between groups. (**B**,**D**) In contrast, the developmental onset was not significantly different.

We performed a bootstrapping hypothesis test^16,17^ with the Benhamini–Hochberg method^18^ to compare the slopes of the regression models. In descending order of developmental pace: groups A4, B4, C4 and D4 (1.43 ± 0.1, 1.12 ± 0.24, 0.82 ± 0.05 and 0.69 ± 0.06; Fig. 2A). The developmental pace of group A4 was comparable to that of group B4 (n = 27, P > 0.1). The developmental pace of group C4 was comparable to that of group D4 (n = 51, P > 0.1). In contrast, the developmental pace of group D4 was significantly slower than those of groups A4 (n = 42, P < 0.01) and B4 (n = 35, P < 0.01). The developmental pace of group C4 was significantly slower than that of group A4 (n = 43, P < 0.02) and showed a trend toward being slower than group B4 (n = 35, P < 0.06).

Next, we compared the estimated onset of neurogenesis from the timing of neural tube closure in humans (4 pcw)^19^ using the bootstrapping hypothesis method^16,17^ with Benhamini–Hochberg method^18^. However, we could not observe significant differences in onset between groups (A4: 11.0 ± 0.8, B4: 11.8 ± 2.5, C4: 10.7 ± 0.6 and D4: 10.5 ± 0.9. A4 vs B4: n = 27, P > 0.8, A4 vs. C4: n = 43, P > 0.7, A4 vs. D4: n = 42, P > 0.7, B4 vs. C4: n = 36, P > 0.8, B4 vs. D4: n = 35, P > 0.8 and C4 vs. D4: n = 51, P > 0.8) (Fig. 2B).

We combined groups A4 and B4 to form group A2 and groups C4 and D4 to form group B2 because neither developmental pace nor onset were significantly different between groups A4 and B4 and between the groups C4 and D4. We confirmed that the developmental pace was significantly different between groups A2 and B2 (A2: 1.3 ± 0.08, B2: 0.78 ± 0.05 and A2 vs. B2: n = 78, P < 0.005) (Fig. 2C). Developmental onset was not significantly different between groups A2 and B2 (A2: 11.2 ± 0.7, B2: 10.3 ± 0.6 and A2 vs. B2; n = 78, P > 0.3) (Fig. 2D). Finally, to rule out the possibility that the combination of developmental events in different brain regions caused differences in developmental paces, we confirmed that the developmental paces were significantly different between brain regions when the regression line was through the predefined onset point (E11 in rat and 4 weeks in human) (Supplementary Figure S2 and Supplementary Table S3).

**Figure 3:**
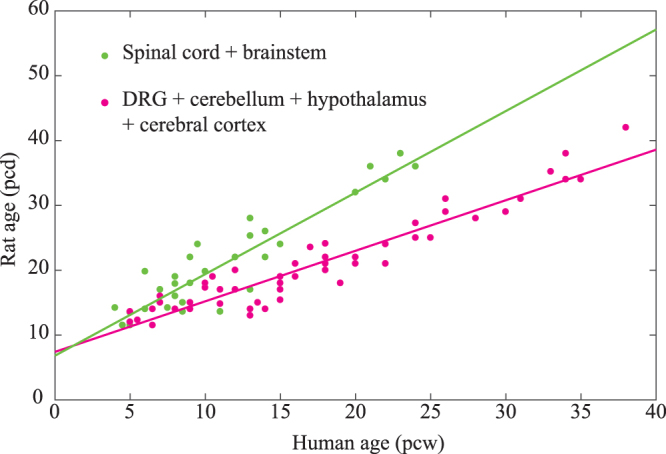
Optimised linear mixed model. We combined pairs (group A4 and B4 and group C4 and D4) because the slope and onset were not significantly different. The filled circles represent developmental timing. The lines indicate the optimised regression line. Green represents a cluster comprising the brainstem and spinal cord. The magenta represents a cluster comprising the DRG, cerebral cortex, cerebellum and hypothalamus.

Consequently, we obtained the following linear mixed model using all developmental events, excluding events 47 and 94 (Fig. 3):

Group 2 A (the spinal cord and brainstem): *rat_pcd = *1.258 × *human_pcw* + 6.832.

Group 2B (the cerebellum, hypothalamus and cortex): *rat_pcd* = 0.774 × *human_pcw* + 7.417.

**Figure 4:**
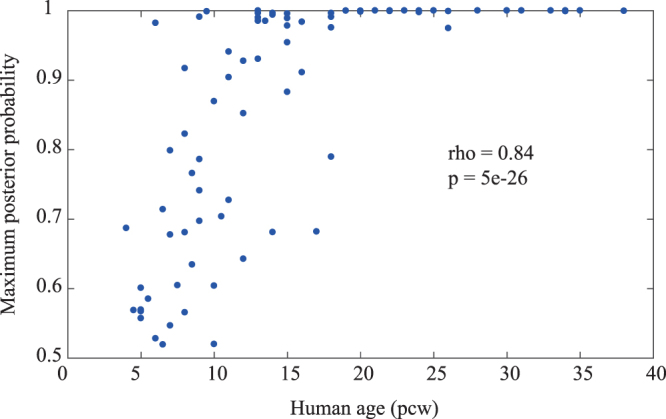
Maximum posterior probability that a developmental event belongs to a cluster correlated with the timing of the event. The filled circle represents a developmental event. The horizontal line represents the timings in the human foetus. The vertical line represents the maximum posterior probability.

Finally, we examined the posterior probability that each developmental event is a member of groups 2 A and B2 (Supplementary Table S2). The group with the maximum posterior probability was not always equal to the group of the event’s corresponding brain region. Because the maximum posterior probability was highly correlated with the timing of developmental events (Spearman’s rank correlation, n = 94, rho = 0.8, P = 5e-25; Fig. 4), these mismatches can be explained by the fact that the classification of each event close to the onset of neurogenesis was difficult. Thus, our classification highly relied on the developmental events during the late human prenatal period.

## Discussion

In the present study, we used a model selection approach to classify brain regions into two developmental groups: 1) spinal cord and brainstem, 2) telencephalon, cerebellum, DRG and hypothalamus. We obtained an optimized linear mixed model using an EM algorithm. This model will provide a translational method between rodents’ and human’s developmental stages, which can be extremely useful when designing animal studies and clinical tests.

Developmental processes are programmed to occur at specific times within individual progenitor cells^20^. Because the timing of developmental events in each brain region can be predicted for rats from human data, using a linear regression model, the majority of developmental timings may be governed by a cell-autonomous mechanism. However, the time-keeping mechanism underlying the comparable developmental paces of mutually separated regions (e.g. the cerebral cortex and cerebellum) remains unknown. An analysis of the spatiotemporal transcriptome^21^ of the brain may reveal that mechanism in future studies.

Previous studies did not identify regional differences in developmental paces^6^, which can be explained by the method of clustering. In the present study, we classified the brain regions using an optimization method. In contrast, in previous studies^5–7^, the limbic system, which includes a broad brain region, was used for analysis. However, developmental paces differed between the brainstem and telencephalon, which indicates that our translational model is better than those previously established because the limbic system consisted of the part of brainstem (the locus ceruleus and raphe), allocortex (hippocampus and amygdala) and hypothalamus in the previous study^6^.

Our model is limited in the human foetal period because the relative developmental pace of postnatal human to rat, estimated previously using the synaptic protein development in visual cortex from birth to adult^22^, was seven-fold slower than the developmental pace of prenatal human to rat in the present study. We removed a few developmental events from our analysis because there were large discrepancies among studies, and we could not determine the precise timing in human (e.g., the onset of olfactory marker protein and the onset of parvalbumin-immunoreactive neurons in the visual cortex). There were a few developmental events for which comparable events could not be identified in rodent (e.g. calcium-binding protein in the inferior olive complex and the onset of synaptophysin in the lateral tuberal hypothalamic nucleus). There were several developmental events we could not use, especially during postnatal development in rat, due to unacceptably large observation error because the interval of juvenile rat age was frequently set to 1 week (e.g. onset of myelination). Developmental events during late gestation had a strong impact on the current results. However, such events were difficult to obtain. Because interactions between different brain regions frequently occur during late gestation, such developmental events may be difficult to translate using our model. Moreover, the chemical cues used in this study were not always cell-type specific markers. We could not rule out the possibility that environmental factors, inter-individual differences and several observation errors due to inter-species differences in the sensitivity of chemical cues affected results. Additional data and further clarification are required in the future. Despite these limitation, our model provides useful information for designing clinical tests on prenatal humans based on rodent data.

## Methods

### Survey of developmental timings

We first conducted an extensive survey on the developmental changes of the human nervous system using the published literature because human data are less abundant than rodent data. We did not use quantitative data because these are difficult to obtain with high accuracy in humans. To classify developmental events by brain regions, the developmental origins must be clearly determined. To discern cell types and developmental origins, our analysis was focused on the onset of chemical markers. We excluded developmental morphological change (i.e. growth of brain region, or synapse formation) from our analysis, if the cell types related with each developmental event could not be identified. As a next step, we searched the literature for comparable developmental sequences with corresponding onsets in the rat brain because availability of comparable developmental events is the highest in rats. No animals were sacrificed in our study. When comparable developmental events could not be identified for rats, we used the corresponding mice data translated by Clancy *et al*.^5^ In such cases, we confirmed the accuracy of mapping by comparing the timing of several developmental events between rats and mice. The translation equation^5^ was represented by the following linear regression model: *rat_day* = 1.24 × *mouse_day* − 1.26. To reduce the measurement error, we restricted events so that the accuracy of onset was less than 3 weeks in humans and 3 days in rats.

To determine the postconceptional day (pcd), we defined the day of insemination as embryonic day (E) 0 in rodents, and whenever the literature used a different method, it was converted into our definition. Postnatal day (P) 0 was defined as E22 for rats. For human, we converted the postconceptional week (pcw) from the gestational week, which was calculated according to the last menses day^23^.

To investigate the developmental paces of each brain region, we subdivided the collected developmental events into the following according to developmental origin: ‘spinal cord’, ‘DRG’, ‘medulla/pons’, ‘cerebellum’, ‘midbrain’, ‘thalamus’, ‘hypothalamus’, ‘subcortex’ (including the basal forebrain and the basal ganglia), ‘allocortex’ (including the hippocampus, amygdala and olfactory bulb), ‘isocortex’ (including the neocortex), ‘retina’ and ‘other’. We determined the region based on the soma position. When the soma position could not be identified, we selected the most plausible position according to the chemical cues. We did not classify the brain regions by functional system (e.g. visual, somatosensory and limbic) because such a classification was not fully supported by molecular mechanisms.

### Model selection

We created a linear mixed model to predict the developmental timings in rats (pcd) based on human developmental timings (pcw). First, we set a group number. Next, the brain regions were classified into one of the groups. The clustering was optimized by an EM algorithm^14^. Because EM algorithms are sensitive to starting values, we randomly searched the best starting values 10,000 times using an AIC criterion^13^. We repeatedly optimized the linear mixed model until the best group number was obtained by an EIC criterion^14^.

### Bootstrapping

We repeatedly resampled *n* developmental events from each group 1,000 times and calculated the regression slope and the estimated timing of neural tube closure. We set *n* to equal the number of data in each group. We conducted a bootstrap hypothesis test following the guidelines^23^. We did not assume equal variances among compared variables. Estimated variances were obtained using an inner bootstrap loop with 50 bootstrap samples. Statistical significance was defined as *p* < 0.05. Multiple comparisons were adjusted by the Benjamini–Hochberg method^18^.

### Data Availability

All data analysed during this study are included in this published article (and its Supplementary Information files).

## Electronic supplementary material

Supplementary Information
