# Supplementary material for: A translational model to determine rodent’s age from human foetal age
Source: Sci Rep. 2017 Dec 8;7:17248. doi: 10.1038/s41598-017-17571-z (PMC5722919; doi:10.1038/s41598-017-17571-z)
Supplement: Supplementary file 1 — Supplementary Information [file 41598_2017_17571_MOESM1_ESM.pdf]

A translational model to determine rodent's age from human foetal age

*Yoshiyuki Ohmura[1], Yasuo Kuniyoshi[1]

[1] Department of Mechano-Informatics, Graduate School of Information Science and Technology, The University of Tokyo 7-3-1, Hongo, Bunkyo-ku, Tokyo, Japan

Table S1 Comparative developmental stages in human and rat.

In the first column of each row, the first line describes a developmental event and the second line describes the related brain region. In the second and third columns, the first line in each row describes the development time which was corrected for comparison, with the original data in brackets, and the second line provides the reference.

Developmental event	Human	Rat
**1 First oligodendrocyte lineage (PDGF-Rα expression) in spinal cord (ventral)**<br>**Spinal cord**	6 (6 pcw)<br>Hajihosseini et al., 1996	14 (E14)<br>Woodruff et al., 2001, Hall et al. 1996
**2 Gliogenetic stage in the ventral spinal cord**<br>**Spinal cord**	4 (6 gw)<br>Marklund et al., 2014	14.2 (E12.5, mouse)<br>Marklund et al., 2014
**3 Motor neurons expressing Er81**<br>**Spinal cord**	8.5 (8.5 pcw)<br>Clowry et al., 2005	13.6 (E12, mouse)<br>Arber et al., 2000
**4 Parvalbumin-positive fibers reach the ventral horn of the cervical segment**<br>**Spinal cord**	8.5 (8.5 pcw)<br>Clowry et al., 2005	15 (E16)<br>Zhang et al., 1990
**5 Olig2- and Pax7-expressing cells derived from dorsal spinal cord**<br>**Spinal cord**	8 (10 gw)<br>Marklund et al., 2014	17.9 (E15.5, mouse)<br>Marklund et al., 2014
**6 Myelination in the cervical spinal cord**<br>**Spinal cord**	10 (10 pcw)<br>Hajihosseini et al., 1996	19.8 (E17, mouse)<br>Foran and Peterson, 1992
**7 Innervation of hindlimb muscle**<br>**Spinal cord**	7 (9 gw)<br>Hesselmans et al., 1993	17 (E17)<br>Reynolds et al., 1991
**8 Elimination of polyneuronal innervation of hindlimb muscle**<br>**Spinal cord**	23 (25 gw)<br>Hesselmans et al., 1993	38 (P16)<br>O'Brein et al., 1978
**9 PGP9.5 fibers penetrate the epidermis**<br>**DRG**	9 (11 gw)<br>Terenghi et al., 1993	15 (E15)<br>Jackman and Fitzgerald, 2000
**10 Presumptive low-threshold mechanoreceptor afferent penetrates the spinal gray matter**<br>**DRG**	13.5 (15.5 gw)<br>Konstantinidou et al., 1995	15 (E15)<br>Jackman and Fitzgerald, 2000
**11 Calcitonin gene-related peptide (CGRP)-immunoreactivity in the DRG**<br>**DRG**	11 (13 gw)<br>Pan et al., 2012, Suburo et al., 1992	17 (E17)<br>Marti et al., 1987, Jackman and Fitzgerald, 2000
**12 CGRP-positive fibers penetrate the epidermis**<br>**DRG**	15 (17 gw)<br>Terenghi et al., 1993	18 (E18)<br>Marti et al., 1987
**13 Substance P-positive fibers in the taste buds**<br>**DRG**	18 (18 pcw)<br>Witt and Reutter, 1998	21 (E21)<br>Yamasaki and Tohyama, 1985
**14 CGRP-positive fibers innervate the heart**<br>**DRG**	16 (18 gw)<br>Gordon et al., 1993	19 (E19)<br>Shoba and Tay, 2000

**15 CGRP-positive fibers prominent in the substantia gelatinosa**<br>**DRG**	22 (24 gw)<br>Marti et al. 1987	21 (E21)<br>Marti et al. 1987
**16 Tyrosine hydroxylase (TH)-positive fibers penetrate the cortical plate**<br>**Medulla/pons**	13 (13 pcw)<br>Zecevic and Verney, 1995	17 (E18)<br>Verney et al., 1984
**17 The first efferent synapse forms below the inner hair cells**<br>**Medulla/pons**	14 (14 pcw)<br>Lavigne-Rebillard and Pujol, 1988	22 (P0)<br>Lenoir et al., 1980
**18 Axo-somatic synapses between the medial efferent and outer hair cells**<br>**Medulla/pons**	22 (22 pcw)<br>Lavigne-Rebillard and Pujol, 1988	34 (P12)<br>Lenoir et al., 1980
**19 5-HT-positive fibers innervate the spinal gray matter**<br>**Medulla/pons**	9 (9 pcw)<br>Sundström et al., 1993	22 (P0)<br>Bregman, 1987
**20 First 5-HT-positive cells**<br>**Medulla/pons**	5 (5 pcw)<br>Sundström et al., 1993	12 (E13)<br>Wallace and Lauder, 1983
**21 First appearance of noradrenergic cells**<br>**Medulla/pons**	5 (5 pcw)<br>Verney et al., 1991,<br>Sundström et al., 1993	11.5 (E12.5)<br>Specht et al., 1981a,<br>Aroca et al., 2006
**22 Diffuse staining of Sonic hedgehog (Shh) in the inner region of the cerebellum**<br>**Cerebellum**	10 (12 gw)<br>Haldipur et al., 2012	17.3, (E15.5, mouse)<br>Haldipur et al., 2012
**23 The first IP3R1-positive cells in the Purkinje cell layer**<br>**Cerebellum**	13 (13 pcw)<br>Milosevic and Zecevic, 1998	14 (E14)<br>Dent et al., 1996
**24 Synapse formation between climbing fibers and Purkinje cells**<br>**Cerebellum**	16 (16 pcw)<br>Zecevic and Rakic, 1976,<br>Milosevic and Zecevic, 1998	19 (E19)<br>Lachamp et al., 2005
**25 First PV-positive Purkinje cells**<br>**Cerebellum**	18 (18 pcw)<br>Milosevic and Zecevic, 1998	20 (E21)<br>Solbach and Celio, 1991
**26 Shh-reactive cells disappear in the external granule layer**<br>**Cerebellum**	18 (20 gw)<br>Haldipur et al., 2012	24.1 (P2, mouse)<br>Haldipur et al., 2012
**27 Young climbing phase in lateral hemisphere of cerebellum**<br>**Cerebellum**	34 (36 gw)<br>Marin-Padilla, 1985	38 (P16)<br>Chedotal and Sotelo, 1992
**28 TH-positive cells in the midbrain**<br>**Midbrain**	4.5 (4.5 pcw)<br>Verney et al., 1991, Almqvist et al., 1996, Pullelles and Verney, 1998	11.5 (E12.5)<br>Specht et al., 1981a
**29 Brn3a-positive cells in the ventral mesencephalon**<br>**Midbrain**	7.5 (7.5 pcw)<br>Nelander et al., 2009	14.2 (E12.5, mouse)<br>Prakash et al., 2009
**30 Catecholamine fibers innervate the habenula region**<br>**Midbrain**	11 (11 pcw)<br>Zecevic and Verney, 1995	17 (E18)<br>Specht et al., 1981b
**31 GAP-43 expression declines in the superior colliculus**<br>**Midbrain**	21 (21 pcw)<br>Qu et al., 2006	36 (P14)<br>Mendonça et al., 2010
**32 TH-positive cells in the zona incerta (A13)**<br>**Thalamus**	5 (5 pcw)<br>Pullelles and Verney, 1998	13.5 (E14.5)<br>Specht et al., 1981a
**33 Calbindin-positive cells and processes in the anteroventral thalamus**<br>**Thalamus**	12 (12 pcw)<br>Kultas-Ilinsky et al., 2004	22 (P0)<br>Frassoni et al., 1991
**34 PV-positive cells in the reticular thalamus**<br>**Thalamus**	12 (12 pcw)<br>Kultas-Ilinsky et al., 2004	22 (P0)<br>Mitrofanis, 1992
**35 GABAergic interneurons in dorsal lateral geniculate nucleus (the dLGN)**<br>**Thalamus**	13 (15 gw)<br>Wadhwa and Bijlani, 1988	25.3 (P3, mouse)<br>Godement et al., 1984
**36 Dendrodendritic contact in dLGN**<br>**Thalamus**	13 (15 gw)<br>Wadhwa and Bijlani, 1988	28 (P6)<br>Aggelopoulos et al., 1989
**37 Thyrotropin-releasing hormone (TRH)-positive cells in the hypothalamus**<br>**Hypothalamus**	8 (8 pcw)<br>Winters et al., 1974	14 (E14)<br>Burgunder and Taylor, 1989
**38 Neurophysin-positive cells in the paraventricular hypothalamus**<br>**Hypothalamus**	12 (14 gw)<br>Mai et al., 1997	17 (E17)<br>Khachaturian and Sladek, 1980
**39 Calbindin-positive cells first appear in the lateral hypothalamus**	13 (15 gw)<br>Koutcherov et al., 2002	13 (E14)<br>Enderlin et al., 1987 ;

**Hypothalamus**		
**40 Somatostatin-positive neurons first appear in the hypothalamus**<br>**Hypothalamus**	14 (16 gw)<br>Chayvialle et al., 1980	14 (E14)<br>Almazan et al. 1989, Shiosaka et al. 1982
**41 Corticotropin-releasing hormone (CRH)-positive cells first appear in the hypothalamus**<br>**Hypothalamus**	15 (17 gw)<br>Bugnon et al., 1982	15.4 (E13.5, mouse)<br>Keegan et al., 1994
**42 Melatonin binding site in the suprachiasmatic nuclei**<br>**Hypothalamus**	15 (17 gw)<br>Thomas et al., 2002	17 (E18)<br>Williams et al., 1991
**43 Neurophysin-positive cells in the suprachiasmatic nuclei**<br>**Hypothalamus**	16 (18 gw)<br>Mai et al., 1997	21 (E18.5, mouse)<br>Silverman et al., 1980, Whitnall et al., 1985
**44 Neuropeptide-Y staining in the arcuate nucleus**<br>**Hypothalamus**	19 (21 gw)<br>Koutcherov et al., 2003	E18 (E18)<br>Foster et al., 1984, Grove et al., 2003
**45 Calbindin-positive mammillothalamic tract fibers penetrate the ventral anterior thalamus**<br>**Hypothalamus**	12 (12 pcw)<br>Kultas-Ilinsky et al., 2004	20 (E20)<br>Puelles et al., 1992, Alppva et al. 2009
**46 Galamin-positive cells in the mammillary nucleus**<br>**Hypothalamus**	25 (27 gw)<br>Bhide and Puranik, 2005	25 (P4)<br>Sizer et al., 1990, Ryan et al., 1997, Burazin et al., 2000
**47 Comparative Arginine vasopressin (AVP)- staining in the suprachiasmatic nuclei**<br>**Hypothalamus**	38 (at term)<br>Swaab et al., 1990	25 (P3)<br>Swaab et al., 1990
**48 Isl1-ir in the lateral ganglionic eminence**<br>**Subcortex**	8 (8 pcw)<br>Onorati et al., 2014	14 (E15)<br>Wang and Liu, 2001
**49 First acetylcholinesterase(AChE)-reactive neurons in the basal forebrain**<br>**Subcortex**	9 (9 pcw)<br>Kostović, 1986	14 (E14)<br>Fine, 1985
**50 External Capsule AChE reactive**<br>**Subcortex**	10.5 (10.5 pcw)<br>Kostović, 1986	19 (E19)<br>De Carlos et al., 1995
**51 AChE-positive fibers penetrate the stratum oriens in the hippocampus**<br>**Subcortex**	24 (24 pcw)<br>Kostović, 1986	25 (P3)<br>Milner et al., 1983
**52 Myelination begin in the caudate-putamen**<br>**Subcortex**	33 (35 gw)<br>Hasegawa et al., 1992	35.2 (P11, mouse)<br>Foran and Peterson, 1992
**53 The secondary dentate matrix forms in the hippocampus**<br>**Allocortex**	11 (11 pcw)<br>Cipriani et al., 2015	17 (E18)<br>Altman and Bayer, 1990
**54 Tbr2-positive Cajal-Retzius cells first appear in the hippocampus**<br>**Allocortex**	11 (11 pcw)<br>Cipriani et al., 2016	14.8 (E13.5, mouse)<br>Hodge et al., 2013
**55 The primary germinal matrix of the dentate gyrus disappears**<br>**Allocortex**	20 (20 pcw)<br>Cipriani et al., 2015	21 (E22)<br>Altman and Bayer, 1990
**56 Calbindin-positive multipolar neurons in the claustrum/amygdala**<br>**Allocortex**	30 (32 gw)<br>Setzer and Ulfig, 1990	29 (P7)<br>Kowiański et al., 2008, 2009
**57 Calbindin immunoreactivity in the str.lucidum along the whole CA3 region except CA3c**<br>**Allocortex**	31 (33gw)<br>Ábrahám et al., 2009	31 (P9)<br>Ábrahám et al., 2007
**58 Calbindin immunoreactivity in the str.lucidum along the whole CA3 region including the CA3c**<br>**Allocortex**	35 (37 gw)<br>Ábrahám et al., 2009	34 (P12)<br>Ábrahám et al., 2007
**59 Anterior commissure fibers cross the midline**<br>**Allocortex**	10 (10 pcw)<br>Rakic and Yakovlev, 1968	18 (E18)<br>Silver et al., 1982, Santacana et al, 1992
**60 Glomeruli formation in the olfactory bulb**<br>**Allocortex**	20 (22 gw)<br>Kharlamova et al., 2015	22 (P0)<br>Treloar et al., 1999
**61 First Reelin-positive cells in the marginal zone**<br>**Isocortex**	5 (5 pcw)<br>Meyer et al., 2000, Bystron et al, 2006	12 (E12)<br>Meyer et al., 1998

**62 Calretinin-positive pioneer cells in the marginal zone**<br>**Isocortex**	6.5 (6.5 pcw)<br>Meyer et al., 2000	11.5 (E11.5)<br>Meyer et al., 1998
**63 First GABAergic neurons in the lateral cortical wall**<br>**Isocortex**	6.5 (6.5 pcw)<br>Meyer et al., 2000	14 (E14)<br>Meyer et al., 1998
**64 DARPP32-positive cells detected in the pallium, but not in the striatum**<br>**Isocortex**	7 (7 pcw)<br>Naimi et al., 1996, Onorati et al., 2014	15 (E15)<br>Foster et al., 1987, Pauly et al., 2013
**65 Cortical plate formation**<br>**Isocortex**	7 (7 pcw)<br>Meyer et al., 2000,<br>Bystron et al. 2006	16 (E16)<br>Weisenhorn et al., 1994,<br>Raedler et al. 1980,<br>VanEden et al. 1989
**66 Callosal fibers cross the midline**<br>**Isocortex**	12 (12 pcw)<br>Rakic and Yakovlev, 1968	17 (E17)<br>Koester and O'Leary, 1994
**67 Er81 or *Er81*-positive layer band in lateral cortex** [a]<br>**Isocortex**	16 (16 pcw)<br>Bayatti et al., 2008	19 (E19)<br>Moroni et al., 2011
**68 Npn1-positive cingulate pioneer axons**<br>**Isocortex**	15 (17 gw)<br>Ren et al., 2006	19 (E19.5)<br>Huang et al., 2012
**69 Ontogeny of KCC2-positive neurons in the cortical plate**<br>**Isocortex**	18 (18 pcw)<br>Sedmak et al., 2015	22 (P0)<br>Clayton et al., 1998
**70 Excitatory GABAergic response in cortical layer I**<br>**Isocortex**	18 (20 gw)<br>Chen and Kriegstein, 2015	22 (P0)<br>Dammerman et al., 2000
**71 Reelin-positive cells below the cortical surface with ascending fibers**<br>**Isocortex**	22 (22 pcw)<br>Meyer et al., 2002	24 (P2)<br>Meyer et al., 1998
**72 Corticospinal neurons innervate cervical spinal motor neurons**<br>**Isocortex**	26 (26 pcw)<br>Eyre et al., 2000	29 (P7)<br>Curfs et al., 1994, Maeda et al., 2016
**73 Mediodorsal thalamus fibers form two intense bands in cortical layer VI**<br>**Isocortex**	28 (28 pcw)<br>Kostović and Goldman-Rakic, 1983	28 (P6)<br>Van Eden, 1986
**74 Switch from bursting to acuity in the light response**<br>**Isocortex**	34 (36 gw)<br>Colonnese et al., 2010	34 (P12)<br>Colonnese et al., 2010
**75 Radial glial processes disappear in the cerebral cortex**<br>**Isocortex**	38 (38 pcw)<br>deAzevedo et al., 2003	42 (P20)<br>Stichel et al., 1991
**76 Nrl or *Nrl* expression in the retina** [b]<br>**Retina**	11 (11 pcw)<br>Hendrickson et al., 2008	13.6 (E12, mouse)<br>Liu et al. 1996,<br>Akimoto et al., 2006
**77 Synaptophysin in the inner plexiform layer**<br>**Retina**	9.5 (11.5 gw)<br>Nag and Wadhwa, 2001	24 (P2)<br>Dhingra et al., 1997
**78 Rod opsin expression in the retina**<br>**Retina**	15 (15 pcw)<br>O'Brein et al., 2003,<br>Hendrickson et al., 2008	24 (P2)<br>Treisman et al., 1988
**79 Synaptophysin in the outer plexiform layer**<br>**Retina**	14 (16 gw)<br>Nag and Wadhwa 2001	26 (P4)<br>Dhingra et al., 1997
**80 Airways are covered with smooth muscle and enveloped by nerve trunks**<br>**Other 1**	5 (7 gw)<br>Sparrow et al., 1990	13.6 (E12, mouse)<br>Sparrow et al., 1990
**81 Open tunnel of Corti**<br>**Other 2**	20 (20 pcw)<br>Lavigne-Rebillard and Pujol, 1988, Pujol et al., 1991	32 (P10)<br>Lenoir et al., 1987
**82 Eyes opening**<br>**Other 3**	24 (26 gw)<br>Duerksen et al., 1994	36 (P14)<br>Colonnese et al., 2010
**83 Gonadotropin-releasing hormone-positive cells first detected in the vomeronasal organ**<br>**Other 4**	5.5 (5.5 pcw)<br>Kjær and Fischer Hansen, 1996, Verney et al., 1996	12.3 (E11, mouse)<br>Schwanzel-Fukuda and Pfaff, 1989
**84 Myelination begins in the optic nerve at chiasm**<br>**Other 5**	30 (32 gw)<br>Magoon amd Robb, 1981,<br>Takayama et al., 1991	29 (P7)<br>Colello et al., 1995
**85 Ossification of maxilla skeleton**<br>**Other 6**	7 (7pcw)<br>Noback et al. 1951	17 (E17)<br>Strong, 1925
**86 Ossification of nasal skeleteon**<br>**Other 6**	9 (9pcw)<br>Noback et al. 1951	18 (E18)<br>Strong, 1925
**87 Ossification of supraoccipital skeleton**	8 (8pcw)<br>Noback et al. 1951	19 (E19)<br>Strong, 1925

**Other 6**		
**88 Merkel cells in the skin**<br>**Other 7**	6 (8 gw)<br>Moll and Moll, 1992, Kim and Holbrook, 1995	19.8 (E17, mouse)<br>Van Keymeulen et al., 2009
**89 Nerve fibers penetrate the tongue epithelium**<br>**Other 8**	8 (8 pcw)<br>Witt and Reutter, 1998	16 (E16)<br>Mbiene and Mistretta, 1997
**90 Neuropeptide Y-positive fibers innervate the heart**<br>**Other 9**	8 (10 gw)<br>Gordon et al., 1993	16 (E16)<br>Shoba and Tay, 2000
**91 Onset of hair follicle bulge**<br>**Other 10**	24 (24 pcw)<br>Narisawa et al., 1993	23.5 (P2, mouse)<br>Nowak et al., 2008
**92 Onset of arrector pili muscles**<br>**Other 11**	24 (24 pcw)<br>Narisawa et al., 1993	27.3 (P5, mouse)<br>Fujiwara et al., 2011
**93 Eyelash growth**<br>**Other 12**	26 (26pcw)<br>Hamming, 1983	31 (P9)<br>Addison et al., 1921
**94 Birth**<br>**Other 13**	38 (38 pcw)<br>Clancy et al., 1991	22 (E22)<br>Clancy et al., 1991

a. The onset of Er81 expression (but not *Er81*) was unknown in rodent. However, the fact that Er81 immunoreactive nuclei were present in the ventricular zone indicates that Er81 expression began earlier than the formation of layer V.

b. The onset of Nrl expression (but not *Nrl*) in the retina was unknown in rodent. However, Nrl is required for the differentiation of rod cells, indicating that the time lag between the onset of Nrl synthesis and that of *Nrl* synthesis is negligible.

Ábrahám H, Orsi H, Seress L (2007) Ontogeny of cocaine- and amphetamine-regulated transcript (CART) peptide and calbindin immunoreactivity in granule cells of the dentate gyrus in the rat. Int J Devl Neurosci 25:265-274.

Ábrahám H, Veszrémi B, Kravják A, Kovács K, Gömöri É, Seress L (2009) Ontogeny of calbindin immunoreactivity in the human hippocampal formation with a special emphasis on granule cells of the dentate gyrus. Int J Devl Neurosci 27:115-127.

Addison, W.H.F. & How, H.W. (1921) The development of the eyelids of the albino rat, until the completion of disjunction. Amer. J. Anat. 29, 1–31.

Aggelopoulos N, Parnavelas JG, Edmunds S(1989) Synaptogenesis in the dorsal lateral geniculate nucleus of the rat. Anat Embryol (Berl) 180:243-257.

Akimoto M, Cheng H, Zhu D, Brzezinski JA, Khanna R, Filippova E, Oh EC, Jing Y, Linares JL, Brooks M, Zareparsi S, Mears AJ, Hero A, Glaser T, Swaroop A (2006) Targeting of GFP to newborn rods by Nrl promoter and temporal expression profiling of flow-sorted photoreceptors. Proc Natl Acad Sci USA 103:3890-3895.

Almazan G, Lefebvre DL, Zingg HH (1989) Ontogeny of hypothalamic vasopressin, oxytocin and somatostatin gene expression. Dev Brain Res 45:69-75.

Almqvist PM, Åkesson E, Wahlberg LU, Pschera H, Seiger Å, Sundsröm E (1996) First trimester development of the human nigrostriatal dopamine system. Exp Neurol 139:227-237.

Alpeeva EV, Makarenko IG (2009) Perinatal development of the mammillothalamic tract and innervation of the anterior thalamic nuclei. Brain Res 1248: 1-13.

Altman J, Bayer SA (1990) Mosaic organization of the hippocampal neuroepithelium and the multiple germinal sources of dentate granule cells. J Comp Neurol 301:325-342.

Arber S, Ladle DR, Lin JH, Frank E, Jessell TM (2000) ETS gene *Er81* controls the formation of functional connections between group Ia sensory afferents and motor neurons. Cell 101:485-498.

Aroca P, Lorente-Cánovas B, Mateos FR, Puelles L (2006) Locus coeruleus neurons originate in alar rhombomere 1 and migrate into the basal plate: Studies in chick and mouse embyros. J Comp Neurol 496:802-818.

Bayatti N, Moss JA, Sun L, Ambrose P, Ward JF, Lindsay S, Clowry GJ (2008) A Molecular neuroanatomical study of the developing human neocortex from 8 to 17 postconceptional weeks revealing the early differentiation of the subplate and subventricular zone. Cereb Cortex 18:1536-1548.

Bhide SA, Puranik SS (2005) Galanin immunohistochemistry and electron microscopic studies in developing human fetal mammillary bodies. J Chem Neuroanat 29:289-295.

Bregman BS (1987) Development of serotonin immunoreactivity in the rat spinal cord and its plasticity after neonatal spinal cord lesions. Brain Res 431:245-263.

Bugnon C, Fellmann D, Bresson JL, Clavequin MC (1982) Immunocytochemical study of the ontogenesis of the CRF-containing neuroglandular system in the human hypothalamus. C R Seances Acad Sci III 294:491-496.

Burazin TC, Larm JA, Ryan MC, Gundlach AL (2000) Galanin-R1 and –R2 receptor mRNA expression during the development of rat brain suggests differential subtype involvement in synaptic transmission and plasticity. Eur J Neurosci 12:2901-2917.

Burgunder J, Taylor T (1989) Ontogeny of Thyrotropin-releasing hormone gene expression in the rat diencephalon. Neuroendocrinology 49:631-640.

Bystron I, Rakic P, Molnár Z, Blakemore C (2006) The first neurons of the human cerebral cortex. Nat Neurosci 9: 880-886.

Chayvialle JA, Paulin C, Dubois PM, Descos F, Dubois MP (1980) Ontogeny of somatostatin in the human gastro-intestinal tract, endocrine pancreas and hypothalamus. Acta Endocrinol (Copenh) 94:1-10.

Chedotal A, Sotelo C (1992) Early development of olivocerebellar projections in the fetal rat using CGRP immunocytochemistry. Eur J Neurosci 4:1159-1179.

Chen J, Kriegstein AR (2015) A GABAergic projection from the zona incerta to cortex promotes cortical neuron development. Science 350:554-558.

Cipriani S, Journiac N, Nardelli J, Verney C, Delezoide AL, Guimiot F, Gressens P, Adle-Biassette H (2015) Dynamic expression patterns of progenitor and neuron layer markers in the developing human dentate gyrus and fimbria. Cereb Cortex bhv223.

Cipriani S, Nardelli J, Verney C, Delezoide AL, Guimiot F, Gressens P, Adle-Biassette H (2016) Dynamic expression patterns of progenitor and pyramidal neuron layer markers in the developing human hippocampus. Cereb Cortex 26:1255-1271.

Clancy, B., Darlington, R.B. & Finlay, B.L. Translating developmental time across mammalian species. Neuroscience 105, 7-17 (2001).

Clayton GH, Owens GC, Wolff JS, Smith RL (1998) Ontogeny of cation-Cl^- cotransporter expression in rat neocortex. Brain Res Dev Brain Res 109:281-292.

Clowry GJ, Moss JA, Clough RL (2005) An immunohistochemical study of the development of sensorimotor components of the early fetal human spinal cord. J Anat 207:313-324.

Colello RJ, Devey LR, Imperato E, Pott U (1995) The Chronology of oligodendrocyte differentiation in the rat optic nerve: Evidence for a signaling step initiating myelination in the CNS. J Neurosci 15:7665-7672.

Colonnese MT, Kaminska A, Minlebaev M, Milh M, Bloem B, Lescure S, Moriette G, Chiron C, Ben-Ari Y, Khazipov R (2010) A conserved switch in sensory processing prepares developing neocortex for vision. Neuron 67:480-498.

Curfs MH, Gribnau AA, Dederen PJ (1994) Selective elimination of transient corticospinal projections in the rat cervical spinal cord gray matter. Brain Res Dev Brain Res 78:182-190.

Dammerman RS, Flint AC, Noctor S, Kriegstein AR (2000) An excitatory GABAergic plexus in developing neocortical layer I. J Neurophysiol 84:428-434.

deAzevedo LC, Fallet C, Moura-Neto V, Daumas-Duport C, Hedin-Pereia C, Lent R (2003) Cortical radial glial cells in human fetuses: Depth-correlated transformation into astrocytes. J Neurobiol 55:288-298.

De Carlos JA, Schlaggar BL, O'Leary DD (1995) Development of acetylcholinesterase-positive thalamic and basal forebrain afferents to embryonic rat neocortex. Exp Brain Res 104:385-401.

Dent MA, Raisman G, Lai FA (1996) Expression of type 1 inositol 1,4,5-trisphosphate receptor during axogenesis and synaptic contact in the central and peripheral nervous system of developing rat. Development 122:1029-1039.

Dhingra NK, Ramamohan Y, Raju TR (1997) Developmental expression of synaptophysin, synapsin I and syntaxin in the rat retina. Brain Res Dev Brain Res 102:267-273.

Duerksen K, Barlow WE, Stasior OG (1994) Fused Eyelids in premature infants. Ophthal Plast Reconstr Surg 10:234-240.

Enderlin S, Norman AW, Celio MR (1987) Ontogeny of the calcium binding protein calbindin D-28K in the rat nervous system. Anat Embryol (Berl) 177:15-28.

Eyre JA, Miller S, Clowry GJ, Conway EA, Watts C (2000) Functional corticospinal projections are established prenatally in the human fetus permitting involvement in the development of spinal motor centres. Brain 123: 51-64.

Fine (1985) Cholinergic basal forebrain development in rat, Neuroscience Letter [Suppl.] 21:72.

Foran DR, Peterson AC (1992) Myelin acquisition in the central nervous system of the mouse revealed by an *MBP-Lac Z* Transgene. J Neurosci 12:4890-4897.

Foster GA & Schultzberg M. (1989) Immunohistochemical analysis of the ontogeny of neuropeptide Y immunoreactive neurons in foetal rat brain. *Int J Dev Neurosci* 2:387-407.

Foster GA, Schultzberg M, Hökfelt T, Goldstein M, Hemmings HC Jr, Ouimet CC, Walaas SI, Greengard P (1987) Development of a dopamine- and cyclic adenosine 3':5'-monophosphate-regulated phosphoprotein (DARPP-32) in the prenatal rat central nervous system, and its relationship to the arrival of presumptive dopaminergic innervation. J Neurosci 7:1994-2018.

Frassoni C, Bentivoglio M, Spreafico R, Sánchez MP, Puelles L, Fairen A (1991) Postnatal development of calbindin and parvalbumin immunoreactivity in the thalamus of the rat. Brain Res Dev Brain Res 58:243-249.

Fujiwara, H. et al. (2011) The basement membrane of hair follicle stem cells is a muscle cell niche. Cell 144, 577–589.

Godement P, Salaün J, Imbert M (1984) Prenatal and postnatal development of retinogeniculate and retiocollicular projections in the mouse. J Comp Neurol 230:552-575.

Gordon L, Polak JM, Moscoso GJ, Smith A, Kuhn DM, Wharton J (1993) Development of the peptidergic innervation of human heart. J Anat 183:131-140.

Grove KL, Smith MS (2003) Ontogeny of the hypothalamic neuropeptide Y system. Physiol Behav 79: 47-63.

Hajihosseini M, Tham TN, Dubois-Dalcq M (1996) Origin of oligodendrocytes within the human spinal cord. J Neurosci 16:7981-7994.

Haldipur P, Bharti U, Govindan S, Sarkar C, Iyengar S, Gressens P, Mani S (2012) Expression of sonic hedgehog during cell proliferation in the human cerebellum. Stem Cells Dev 21:1059-1068.

Hall A, Giese NA, Richardson WD (1996) Spinal cord oligodendrocytes develop from ventrally derived progenitor cells that express PDGF alpha-receptors. Development 122: 4085-4094.

Hamming, N. (1983) Anatomy and embryology of the eyelids: a review with special reference to the development of divided nevi. Peiatr. Dermatol**.** 1, 51–58.

Hasegawa M, Houdou S, Mito T, Takashima S, Asanuma K, Ohno T (1992) Development of myelination in the human fetal and infant cerebrum: A myelin basic protein immunohistochemical study. Brain Dev 14:1-6.

Hendrickson A, Bumsted-O'Brien K, Natoli R, Ramamurthy V, Possin D, Provis J (2008) Rod photoreceptor differentiation in fetal and infant human retina. Exp Eye Res 87:415-426.

Hesselmans LF, Jennekens FG, Van den Oord CJ, Veldman H, Vincent A (1993) Development of innervation of skeletal muscle fibers in man: Relation to acetylcholine receptors. Anat Rec 236:553-562.

Hodge RD, Garcia AJ III, Elsen GE, Nelson BR, Mussar KE, Reiner SL, Ramirez JM, Hevner RF (2013) *Tbr2* expression in Cajal-Retzius cells and intermediate neuronal progenitors is required for morphogenesis of the dentate gyrus. J Neurosci 33:4165-4180.

Huang CY, Chu D, Hwang WC, Tsaur ML (2012) Coexpression of high-voltage-activated ion channels Kv3.4 and Cav1.2 in pioneer axons during pathfinding in the developing rat forebrain. J Comp Neurol 520:3650-3672.

Jackman A, Fitzgerald M (2000) Development of peripheral hindlimb and central spinal cord innervation by subpopulations of dorsal root ganblion cells in the embryonic rat. J Comp Neurol 418:281-298.

Keegan CE, Herman JP, Karolyi IJ, O'Shea KS, Camper SA, Seasholtz AF (1994) Differential expression of corticotropin-releasing hormone in developing mouse embryos and adult brain. Endocrinology 134:2547-2555.

Khachaturian H, Sladek JR Jr (1980) Simultaneous monoamine histofluorescence and neuropeptide immunocytochemistry: III. Ontogeny of catecholamine varicosities and neurophysin neurons in the rat supraoptic and paraventricular nuclei. Peptide 1:77-95.

Kharlamova AS, Barabanov VM, Saveliev SV (2015) Development of human olfactory bulbs in prenatal ontogenesis: An immunohistochemical study with markers of presynaptic terminals (Anti-SNAP-25, Synapsin-I, and Synaptophysin). Ontogenez 46:174-185.

Kim DK, Holbrook KA (1995) The appearance, density, and distribution of Merkel cells in human embryonic and fetal skin: Their relation to sweat gland and hair follicle development. J Invest Dermatol 104:411-416.

Kjær I, Fischer Hansen B(1996) The human vomeronasal organ: prenatal developmental stages and distribution of luteinizing hormone-releasing hormone. Eur J Oral Sci 104:34-40.

Koester SE, O'Leary DD (1994) Axons of early generated neurons in cingulate cortex pioneer the corpus callosum. J Neurosci 14: 6608-6620.

Konstantinidou AD, Silos-Santiago I, Flaris N, Snider WD (1995) Development of primary afferent projection in human spinal cord. J Comp Neurol 354:1-12.

Kostović I (1986) Prenatal development of nucleus basalis complex and related fiber systems in man: A histochemical study. Neuroscience 17:1047-1077.

Koutcherov Y, Mai JK, Ashwell KW, Paxions G (2002) Organization of human hypothalamus in fetal development. J Comp Neurol 446:301-324.

Kowiański P, Dziewiątkowski J, Moryś JM, Majak K, Wójcik S, Edelstein LR, Lietzau G, Moryś J (2009) Colocalization of neuropeptides with calcium-binding proteins in the claustral interneurons during postnatal development of the rat. Brain Res Bull 80:100-106.

Kowiański P, Moryś JM, Dziewiątkowski J, Wójcik S, Sidor-Kaczmarek J, Moryś J (2008) NPY-, SOM- and VIP-containing interneurons in postnatal development of the rat claustrum. Brain Res Bull 76:565-571.

Kostović I, Goldman-Rakic PS (1983) Transient Cholinesterase staining in the mediodorsal nucleus of the thalamus and its connections in the developing human and monkey brain. J Comp Neurol 219:431-447.

Kultas-Ilinsky K, Fallet C, Verney C (2004) Development of the human motor-related thalamic nuclei during the first half of gestatin, with special emphasis on GABAergic circuits. J Comp Neurol 476:267-289.

Lachamp P, Balland B, Tell F, Baude A, Strube C, Crest M, Kessler JP (2005) Early expression of AMPA receptors and lack of NMDA receptors in developing rat climbing fibre synapses. J Physiol 564:751-763.

Lavigne-Rebillard M, Pujol R (1988) Hair cell innervation in the fetal human cochlea. Acta Otolaryngol 105:398-402.

Lenoir M, Puel JL, Pujol R (1987) Stereocilia and tectorial membrane development in the rat cochlea. A SEM study. Anat Embryol (Berl) 175:477-487.

Lenoir M, Shnerson A, Pujol R (1980) Cochlear receptor development in the rat with emphasis on synaptogenesis. Anat Embryol (Berl) 160:253-262.

Liu Q, Ji X, Breitman ML, Hitchcock PF, Swaroop A (1996) Expression of the bZIP transcription factor gene Nrl in the developing nervous system. Oncogene 12: 207-211.

Maeda H, Fukuda S, Kameda H, Murabe N, Isoo N, Mizukami H, Ozawa K, Sakurai M (2016) Corticospinal axons make direct synaptic connections with spinal motoneurons innervating forearm muscles early during postnatal development in the rat. J Physiol 594:189-205.

Magoon EH, Robb RM (1981) Development of myelin in human optic nerve and tract: A light and electron microscopic study. Arch Ophthalmol 99:655-659.

Mai JK, Lensing-Höhn S, Ende AA, Sofroniew MV (1997) Developmental organization of neurophysin neurons in the human brain. J Comp Neurol 385:477-489.

Marin-Padilla M (1985) Neurogenesis of the climbing fibers in the human cerebellum: A Golgi study. J Comp Neurol 235:82-96.

Marklund U, Alekseenko Z, Andersson E, Falci S, Westgren M, Perlmann T, Graham A, Sundström, Ericson J (2014) Detailed expression analysis of regulatory genes in the early developing human neural tube. Stem Cells Dev 23:5-15.

Marti E, Gibson SJ, Polak JM, Facer P, Springall DR, Van Aswegen G, Aitchison M, Koltzenburg M (1987) Ontogeny of peptide- and amine-containing neurons in motor, sensory, and autonomic regions of rat and human spinal cord, dorsal root ganglia, and rat skin. J Comp Neurol 266:332-359.

Mbiene JP, Mistretta CM (1997) Initial innervation of embryonic rat tongue and developing taste papillae: Nerves follow distinctive and spatially restricted pathways. Acta Anat (Basel) 160:139-158.

Mendonça HR, Araújo SE, Gomes AL, Sholl-Franco A, da Cunha Faria Melibeu A, Serfaty CA, Campello-Costa P (2010) Expression of GAP-43 during development and after monocular enucleation in the rat superior colliculus. Neurosci Lett 477:23-27.

Meyer G, Perez-Garcia CG, Abraham H, Caput D (2002) Expression of p73 and reelin in the developing human cortex. J Neurosci 22:4973-4986.

Meyer G, Schaaps JP, Moreau L, Goffinet AM (2000) Embryonic and early fetal development of the human neocortex. J Neurosci 20:1858-1868.

Meyer G, Soria JM, Martínez-Galán JR, Martín-Clemente B, Fairén A (1998) Different origins and developmental histories of transient neurons in the marginal zone of the fetal and neonatal rat cortex. J Comp Neurol 397:493-518.

Milner TA, Loy R, Amaral DG (1983) An anatomical study of the development of the septo-hippocampal projection in the rat. Brain Res 284:343-371.

Milosevic A, Zecevic N (1998) Developmental changes in human cerebellum: Expression of intracellular calcium receptors, calcium-binding proteins, and phosphorylated and nonphosphorylated neurofilament protein. J Comp Neurol 396:442-460.

Mitrofanis J (1992) Patterns of antigenic expression in the thalamic reticular nucleus of developing rat. J Comp Neurol 320:161-181.

Moll I, Moll R (1992) Early development of human Merkel cells. Exp Dermatol 1:180-184.

Moroni RF, Cipelletti B, Inverardi F, Regondi MC, Spreafico R, Frassoni C (2011) Development of cortical malformations in BCNU-treated rat, model of cortical dysplasia. Neuroscience 175:380-393.

Nag TC, Wadhwa S (2001) Differential expression of syntaxin-1 and synaptophysin in the developing and adult human retina. J Biosci 26:179-191.

Naimi S, Jeny R, Hantraye P, Peschanski M, Riche D (1996) Ontogeny of human striatal DARPP-32 neurons in fetuses and following Xenografting to the adult rat brain. Exp Neurol 137:15-25.

Narisawa, Y., Hashimoto, K., Nakamura, Y. & Kohda, H. (1993) A high concentration of Merkel cells in the bulge prior to the attachment of the arrector pili muscle and the formation of the perifollicular nerve plexus in human fetal skin. Arch Dertamtol. Res. 285, 261–268.

Nelander J, Hebsgaard JB, Parmar M (2009) Organization of the human embryonic ventral mesencephalon. Gene Expr Patterns 9:555-561.

Noback, C.R. & Robertson, G.G. (1951) Sequences of appearance of ossification centers in the human skeleton during the first five prenatal months. Am. J. Anat. 89: 1–28.

Nowak, J.A., Polak, L., Pasolli, H.A. & Fuchs, E. (2008) Hair follicle stem cells are specified and function in early skin morphogenesis. Cell Stem Cell 3, 33–43.

O'Brien RA, Östberg AJ, Vrbová G (1978) Observations on the elimination of polyneuronal innervation in developing mammalian skeletal muscle. J Physiol. 282:571-582.

O'Brien KM, Schulte D, Hendrickson AE (2003) Expression of photoreceptor-associated molecules during human fetal eye development. Mol Vis 9:401-409.

Onorati M, Castiglioni V, Biasci D, Cesana E, Menon R, Vuono R, Talpo F, Laguna Goya R, Lyons PA, Bulfamante GP, Muzio L, Martino G, Toselli M, Farina C, Barker RA, Biella G, Cattaneo E (2014) Molecular and functional definition of the developing human striatum. Nat Neurosci 17:1804-1815.

Pan A, Wu H, Li M, Lu D, He X, Yi X, Yan XX, Li Z (2012) Prenatal expression of purinergic receptor P2X3 in human dorsal root ganglion. Purinergic Signal 8:245-254.

Prakash N, Puelles E, Freude K, Trümbach D, Omodei D, Di Salvio M, Sussel L, Ericson J, Sander M, Simeone A, Wurst W (2009) Nkx6-1 controls the identity and fate of red nucleus and oculomotor neurons in the mouse midbrain. Development 136:2545-2555.

Pauly MC, Döbrössy MD, Nikkhah G, Winkler C, Piroth T(2013) Organization of the human fetal subpallium. Front Neuroanat 7:54.

Puelles L, Sánchez MP, Spreafico R, Fairén A (1992) Prenatal development of calbindin immunoreactivity in the dorsal thalamus of the rat. Neuroscience 46:135-147.

Puelles L, Verney C (1998) Early neuromeric distribution of tyrosine-hydroxylase-immunoreactive neurons in human embryos. J Comp Neurol 394:283-308.

Pujol R, Lavigne-Rebillard M, Uziel A (1991) Development of the human cochlea. Acta Otolaryngol Suppl 482:7-12.

Qu J, Zhou X, Zhu H, Cheng G, Ashwell KW, Lu F (2006) Development of the human superior colliculus and the retinocollicular projection. Exp Eye Res 82:300-310.

Rakic P, Yakovlev PI (1968) Development of the corpus callosum and cavum septi in Man. J Comp Neurol 132: 45-72.

Raedler E, Raedler A, Feldhaus S (1980) Dynamical aspects of neocortical histogenesis in the rat. Anat Embryol (Berl) 158: 253-269.

Ren T, Anderson A, Shen WB, Huang H, Plachez C, Zhang J, Mori S, Kinsman SL, Richards LJ (2006) Imaging, anatomical, and molecular analysis of callosal formation in the developing human fetal brain. Anat Rec A Discov Mol Cell Evol Biol 288:191-204.

Reynolds ML, Fitzgerald M, Benowitz LI (1991) GAP-43 expression in developing cutaneous and muscle nerves in the rat hindlimb. Neuroscience 41:201-211.

Ryan MC, Loiacono RE, Gundlach AL (1997) Galamin messenger RNA during postnatal development of the rat brain: expression patterns in Purkinje cells differentiate anterior and posterior lobes of cerebellum. Neuroscience 78:1113-1127.

Santacana M, Heredia M, Valverde F (1992) Development of the main efferent cells of the olfactory bulb and the bulbar component of the anterior commissure. Brain Res Dev Brain Res 65:75-83.

Schwanzel-Fukuda M, Pfaff DW (1989) Origin of luteinizing hormone-releasing hormone neurons. Nature 338:161-164.

Sedmak G, Jovanov-Milošević N, Puskarjov M, Ulamec M, Krušlin B, Kaila K, Judaš M (2015) Developmental expression patterns of KCC2 and functionally associated molecules in the human brain. Cereb Cortex:bhv218.

Setzer M, Ulfig N (1999) Differential expression of calbindin and calretinin in the human fetal amygdala. Microsc Res Tech 46:1-17.

Shiosaka S, Takatsuki K, Sakanaka M, Inagaki S, Takagi H, Senba E, Kawai Y, Iida H, Minagawa H, Hara Y, Matsuzaki T, Tohyama M (1982) Ontogeny of somatostatin-containing neuron system of the rat: Immunohistochemical analysis. II. Forebrain and Diencephalon. J Comp Neurol 204:211-224.

Shoba T, Tay SS (2000) Nitrergic and peptidergic innervation in the developing rat heart. Anat Embryol (Berl) 201:491-500.

Silver J, Lorenz SE, Wahlsten D, Coughlin J (1982) Axonal guidance during development of the great cerebral commissures: Descriptive and experimental studies, in vivo, on the role of preformed glial pathways. J Comp Neurol 210:10-29.

Silverman AJ, Goldstein R, Gadde CA (1980) The ontogenesis of neurophysin-containing neurons in the mouse hypothalamus. Peptides 1 Suppl 1:27-44.

Sizer AR, Rökaeus A, Foster GA (1990) Analysis of the ontogeny of galanin in the rat central nervous system by immunohistochemistry and radioimmunoassay. Int J Devl Neuroscience 8:81-97.

Solbach S Celio MR (1991) Ontogeny of the calcium binding protein parvalbumin in the rat nervous system. Anat Embryol (Berl) 184:103-124.

Sparrow MP, Weichselbaum M, McCray PB (1990) Development of the innervation and airway smooth muscle in human fetal lung. Am J Respir Cell Mol Biol 20:550-560.

Specht LA, Pickel VM, Joh TH, Reis DJ (1981a) Light-microscopic immunocytochemical localization of tyrosine hydroxylase in prenatal rat brain. I. Early ontogeny. J Comp Neurol 199:233-253.

Specht LA, Pickel VM, Joh TH, Reis DJ (1981b) Light-microscopic immunocytochemical localization of tyrosine hydroxylase in prenatal rat brain. II. Late ontogeny. J Comp Neurol 199:255-276.

Stichel CC, Müller CM, Zilles K (1991) Distribution of glial fibrillary acidic protein and vimentin immunoreactivity during rat visual cortex development. J Neurocytol 20:97-108.

Strong, R.M. (1925) The order, time, and rate of ossification of the albino rat (Mus Norvegicus Albinus) skeleton. Am. J. Anat. 36, 213–355.

Suburo AM, Gu XH, Moscoso G, Ross A, Terenghi G, Polak JM (1992) Developmental pattern and distribution of nerve growth factor low-affinity receptor immunoreactivity in human spinal cord and dorsal root ganglia: Comparison with synaptophysin, neurofilament and neuropeptide immunoreactivities. Neuroscience 50:467-482.

Sundström E, Kölare S, Souverbie F, Samuelsson EB, Pschera H, Lunell NO, Seiger A (1993) Neurochemical differentiation of human bulbospinal monoamine neurons during the first trimester. Brain Res Dev Brain Res 75:1-12.

Swaab DF, Hofman MA, Honnebier MB (1990) Development of vasopressin neurons in the human suprachiasmatic nucleus in relation to birth. Brain Res Dev Brain Res 53:289-293.

Takayama S, Yamamoto M, Hashimoto K, Itoh H (1991) Immunohistochemical study on the developing optic nerves in human embryos and fetuses. Brain Dev 13:307-312.

Terenghi G, Sundaresan M, Moscoso G, Polak JM (1993) Neuropeptides and a neuronal marker in cutaneous innervation during human foetal development. J Comp Neurol 328:595-603.

Thomas L, Purvis CC, Drew JE, Abramovich DR, Williams LM (2002) Melatonin receptors in human fetal brain:2-[^{125}I]iodomelatonin binding and MT1 gene expression. J Pineal Res 33:218-224.

Treisman JE, Morabito MA, Barnstable CJ (1988) Opsin expression in the rat retina is developmentally regulated by transcriptional activation. Mol Cell Biol 8: 1570-1579.

Treloar HB, Purcell AL, Greer CA (1999) Glomerular formation in the developing rat olfactory bulb. J Comp Neurol 413:289-304.

Van Eden CG (1986) Development of connections between the mediodorsal nucleus of the thalamus and the prefrontal cortex in the rat. J Comp Neurol 244:349-359.

VanEden CG, Mrzljak L, Voorn P, Uylings HBM (1989) Prenatal development of GABAergic neurons in the neocortex of the rat. J Comp Neuol 289:213-227.

Van Keymeulen A, Mascre G, Youseff KK, Harel I, Michaux C, De Geest N, Szpalski C, Achouri Y, Bloch W, Hassan BA, Blanpain C (2009) Epidermal progenitors give rise to Merkel cells during embryonic development and adult homeostasis. J Cell Biol 187:91-100.

Verney C, Berger B, Baulac M, Helle KB, Alvarez C (1984) Dopamine-β-hydroxylase-like immunoreactivity in the fetal cerebral cortex of the rat: Noradrenergic ascending pathways and terminal fields. Int J Devl Neurosci 12:491-503.

Verney C, el Amraoui A, Zecevic N (1996) Comigration of tyrosine hydroxylase- and gonadotropin-releasing hormone-immunoreactive neurons in the nasal area of human embryos. Brain Res Dev Brain Res 97:251-259.

Verney C, Zecevic N, Nikolic B, Alvarez C, Berger B (1991) Early evidence of catecholaminergic cell groups in 5- and 6-week-old human embryos using tyrosine hydroxylase and dopamine-ß-hydroxylase immunocytochemistry. Neurosci Lett 131:121-124.

Wadhwa S, Bijlani V (1988) Cytodifferentiation and developing neuronal circuitry in the human lateral geniculate nucleus. Int J Devl Neuroscience 6:59-75.

Wallace JA, Lauder JM (1983) Development of the serotonergic system in the rat embryo: An immunocytochemical study. Brain Res Bull 10: 459-479.

Wang HF, Liu FC (2001) Developmental restriction of the LIM homeodomain transcription factor Islet-1 expression to cholinergic neurons in the rat striatum. Neuroscience 103: 999-1016.

Weisenhorn DM, Prieto EW, Celio M (1994) Localization of calretinin in cells of layer I (Cajal-Retzius cells) of the developing cortex of rat. Brain Res Dev Brain Res 82:293-297.

Whitnall MH, Key S, Ben-Barak Y, Ozato K, Gainer H. (1985) Neurophysin in the hypothalamo-neurohypophysial system II. Immunocytochemical studies of the ontogeny of Oxytocinergic and Vasopressinergic neurons. J Neurosci 5:98-109

Williams LM, Martinoli MG, Titchener LT, Pelletier G (1991) The ontogeny of central melatonin binding sites in the rat. Endocrinology 128: 2083-2090.

Winters AJ, Eskay RL, Porter JC (1974) Concentration and distribution of TRH and LRH in the human fetal brain. J Clin Endocrinol Metab 39: 960-963.

Witt M, Reutter K (1998) Innervation of developing human taste buds. An immunohistochemical study. Histochem Cell Biol 109:281-291.

Woodruff RH, Tekki-Kessaris N, Stiles CD, Rowitch DH, Richardson WD (2001) Oligodendrocyte development in the spinal cord and telencephalon: Common themes and new perspectives. Int J Dev Neurosci 19:379-385.

Yamasaki H, Tohyama M (1985) Ontogeny of substance P-like immunoreactive fibers in the taste buds and their surrounding epithelium of the circumvallate papillae of the rat. II. Electron microscopic analysis. J Comp Neurol 241:493-502.

Zeceivc N, Rakic P (1976) Differentiation of Purkinje cells and their relationship to other components of developing cerebellar cortex in man. J Comp Neurol 167:27-48.

Zecevic N, Verney C (1995) Development of the catecholamine neurons in human embryos and fetuses, with special emphasis on the innervation of the cerebral cortex. J Comp Neurol 351:509-535.

Zhang JH, Morita Y, Hironaka T, Emson PC, Tohyama M (1990) Ontological study of calbindin-D_{28k}-like and parvalbumin-like immunoreactivities in rat spinal cord and dorsal root ganglia. J Comp Neurol 302:715-728.

Table S2 Posterior probability

In the first column, each line describes a developmental event number. The second column indicates the related brain region. The third column indicates the clustering result. In the fourth and fifth columns, each row describes the posterior probability that each developmental event is member of cluster A2 and B2, respectively. Values in bold represent the maximum posterior probability within the two clusters.

events	Brain region	Cluster	A2	B2
1	Spinal cord	A2	**0.528213**	0.471787
2			**0.687014**	0.312986
3			0.234027	**0.765973**
4			0.365485	**0.634515**
5			**0.822613**	0.177387
6			**0.869444**	0.130556
7			**0.798697**	0.201303
8			**1**	1.32E-07
9	DRG	B2	0.302775	**0.697225**
10			0.014966	**0.985034**
11			0.272614	**0.727386**
12			0.021774	**0.978226**
13			0.009126	**0.990874**

14			0.016288	**0.983712**
15			3.46E-05	**0.999965**
16	Medulla/pons	A2	0.069565	**0.930435**
17			**0.681128**	0.318872
18			**0.999856**	0.000144
19			**0.990945**	0.009055
20			0.430463	**0.569537**
21			0.399	**0.601**
22	Cerebellum	B2	0.479966	**0.520034**
23			0.014835	**0.985165**
24			0.016288	**0.983712**
25			0.003656	**0.996344**
26			0.210379	**0.789621**
27			0.001007	**0.998993**
28	Midbrain	A2	0.431123	**0.568877**
29			0.395287	**0.604713**
30			0.272614	**0.727386**
31			**1**	4.77E-07
32	Thalamus	A2	**0.557258**	0.442742
33			**0.927547**	0.072453
34			**0.927547**	0.072453
35			**0.996396**	0.003604
36			**0.999925**	7.54E-05
37	Hypothalamus	B2	0.319096	**0.680904**
38			0.147873	**0.852127**
39			0.010266	**0.989734**
40			0.006063	**0.993937**
41			0.004375	**0.995625**
42			0.011057	**0.988943**
43			0.088871	**0.911129**
44			0.000207	**0.999793**
45			**0.642735**	0.357265
46			1.99E-05	**0.99998**
47				
48	Subcortex	B2	0.319096	0.680904
49			0.213924	0.786076
50			0.703786	0.296214
51			0.000113	0.999887
52			2.65E-05	0.999974
53	Allocortex	B2	0.272614	**0.727386**
54			0.095983	**0.904017**
55			0.000651	**0.999349**
56			3.16E-07	**1**
57			9.53E-07	**0.999999**
58			1.78E-08	**1**
59			**0.604047**	0.395953
60			0.001758	**0.998242**
61	Isocortex	B2	0.430463	**0.569537**
62			0.286023	**0.713977**
63			0.480574	**0.519426**
64			0.546804	0.453196
65			**0.677573**	0.322427

66			0.147873	**0.852127**
67			0.016288	**0.983712**
68			0.045833	**0.954167**
69			0.024441	**0.975559**
70			0.024441	**0.975559**
71			0.00085	**0.99915**
72			0.000943	**0.999057**
73			4.32E-06	**0.999996**
74			2.14E-07	**1**
75			0.000483	**0.999517**
76	Retina	A2	0.059183	**0.940817**
77			**0.998752**	0.001248
78			**0.88286**	0.11714
79			**0.996402**	0.003598
80	Airways	A2	**0.567214**	0.432786
81	Tunnel of Corti	A2	**0.999675**	0.000325
82	Eyelid	A2	**0.999937**	6.30E-05
83	Vomeronasal organ	B2	0.414782	**0.585218**
84	Optic nerve	B2	3.16E-07	**1**
85	Skeleteon	A2	**0.798697**	0.201303
86			**0.741106**	0.258894
87			**0.917013**	0.082987
88	Skin	A2	**0.982123**	0.017877
89	Tongue	A2	**0.565683**	0.434317
90	Heart	A2	**0.565683**	0.434317
91	Hair	B2	0.317986	**0.682014**
92	Skin	B2	0.002391	**0.997609**
93	Hair	B2	0.025365	**0.974635**
94	Birth			

Possible translational model of the hypothalamus

In the current study, we did not use developmental events 47 and 94 for analysis because we could not rule out the possibility that these birth-related events were outliers. When developmental event 47 was used for constructing a translational model of the hypothalamus, the nonlinear model was better predictor than the linear model by AIC criterion (Supplementary Figure S1). To judge whether birth-related developmental events were real outliers, future studies will require additional comparative developmental events in the hypothalamus, especially during the late foetal period.

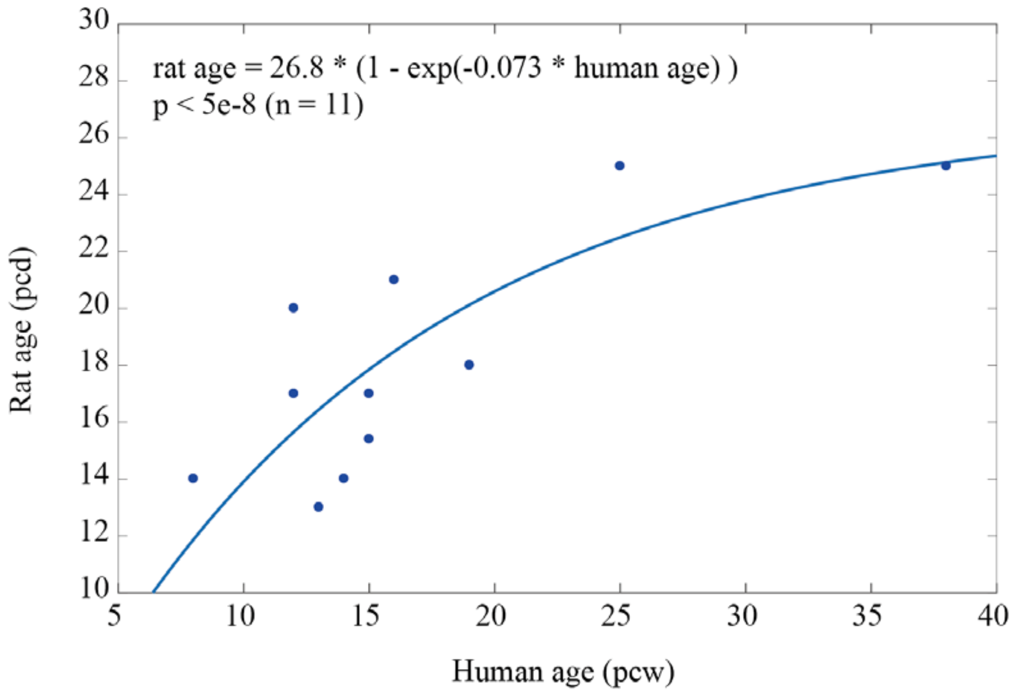

Supplementary Figure S1. Possible translational model of the development of the hypothalamus. Filled circles represent developmental timing. The line represents the optimised non-linear model.

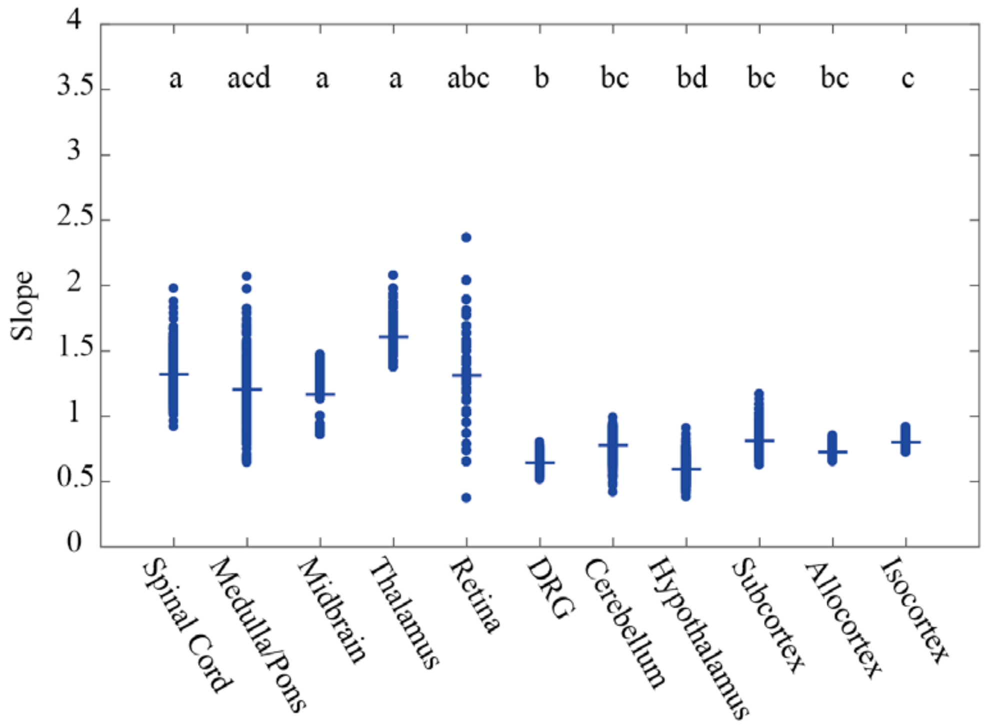

Supplementary Figure S2. Bootstrap replications of the slope of regression lines through onset points (4 weeks in human and 11 days in rat). Different letters represent significant differences.

Supplementary Table S3. P-value of bootstrapping hypothesis test.

Each line indicates the p-value of a bootstrapping hypothesis test with the Benjamini-Hochberg adjustment, the null hypothesis was that the regression slope of the brain region in a column and its corresponding row are drawn from the same population. We assumed that the regression line was through the onset point (4 week in human and E11 in rat). Each line in brackets represents p-value before multi-compare adjustment. Bold line represents $p < 0.05$. SC = spinal cord (n = 8), MP = medulla/pons (n = 6), MB = midbrain (n = 4), TH = thalamus (n = 5), Rt = Retina (n = 4), DRG = DRG (n = 7), CR = Cerebellum (n = 6), HY = Hypothalamus (n = 10), Su = Subcortex (n = 8), AC = Allocortex (n = 8), IC = Isocortex (n = 15).

Group B2					Group A2						
AC	Su	HY	CR	DRG	Rt	TH	MB	MP	SC		
									P=0.204 (P=0.152)	MP	Group A2
								P=0.751 (P=0.669)	P=0.514 (P=0.43)	MB	
							P=0.427 (P=0.342)	P=0.134 (P=0.080)	P=0.831 (P=0.801)	TH	
						P=0.411 (P=0.322)	P=0.965 (P=0.965)	P=0.803 (P=0.747)	P=0.493 (P=0.404)	Rt	
					P=0.090 (**P=0.044**)	**P=0.006 (P=0.002)**	**P=0.006 (P=0.002)**	**P=0.042 (P=0.016)**	**P=0.006 (P=0.002)**	DRG	Group B2
				P=0.156 (P=0.108)	P=0.143 (P=0.096)	**P=0.006 (P=0.002)**	**P=0.006 (P=0.002)**	P=0.102 (P=0.054)	**P=0.006 (P=0.002)**	CR	
			P=0.078 (**P=0.034**)	P=0.627 (P=0.535)	P=0.090 (**P=0.044**)	**P=0.006 (P=0.002)**	**P=0.006 (P=0.002)**	P=0.065 (**P=0.026**)	**P=0.006 (P=0.002)**	HY	
		P=0.090 (**P=0.046**)	P=0.803 (P=0.759)	P=0.128 (P=0.07)	P=0.176 (P=0.128)	**P=0.006 (P=0.002)**	**P=0.006 (P=0.002)**	P=0.140 (P=0.088)	**P=0.006 (P=0.002)**	Su	
	P=0.385 (P=0.294)	P=0.140 (P=0.084)	P=0.648 (P=0.565)	P=0.140 (P=0.092)	P=0.090 (**P=0.042**)	**P=0.006 (P=0.002)**	**P=0.006 (P=0.002)**	P=0.076 (**P=0.032**)	**P=0.006 (P=0.002)**	AC	
P=0.14 (P=0.092)	P=0.873 (P=0.857)	**P=0.022 (P=0.008)**	P=0.803 (P=0.749)	**P=0.035 (P=0.002)**	P=0.176 (P=0.128)	**P=0.006 (P=0.002)**	**P=0.006 (P=0.002)**	P=0.134 (P=0.076)	**P=0.006 (P=0.002)**	IC	
